# Washing effect on superparamagnetic iron oxide nanoparticles

**DOI:** 10.1016/j.dib.2016.03.104

**Published:** 2016-04-07

**Authors:** Laura-Karina Mireles, Edward Sacher, L’Hocine Yahia, Sophie Laurent, Dimitri Stanicki

**Affiliations:** a Laboratoire d’Innovation et d’Analyse de Bioperformance, École Polytechnique de Montréal, C.P. 6079, Succursale Centre-ville, Montréal, Québec, Canada H3C 3A7; b Département de Génie physique, École Polytechnique de Montréal, C.P. 6079, Succursale Centre-ville, Montréal, Québec, Canada H3C 3A7; c Department of General, Organic, Biomedical Chemistry, NMR and Molecular Imaging Laboratory, Université de Mons, 19 Avenue Maistriau, B-7000 Mons, Belgium; d Center for Microscopy and Molecular Imaging (CMMI), B-6041 Gosselies, Belgium

**Keywords:** Superparamagnetic iron oxide nanoparticles, Washing effect, Surface chemistry, Prodrugs, Dialysis effect

## Abstract

Much recent research on nanoparticles has occurred in the biomedical area, particularly in the area of superparamagnetic iron oxide nanoparticles (SPIONs); one such area of research is in their use as magnetically directed prodrugs. It has been reported that nanoscale materials exhibit properties different from those of materials in bulk or on a macro scale [1]. Further, an understanding of the batch-to-batch reproducibility and uniformity of the SPION surface is essential to ensure safe biological applications, as noted in the accompanying article [2], because the surface is the first layer that affects the biological response of the human body. Here, we consider a comparison of the surface chemistries of a batch of SPIONs, before and after the supposedly gentle process of dialysis in water.

## **Specifications table**

**Table:** 

Subject area	Chemistry, Physics, Biology
More specific subject area	Surface characterization
Type of data	Table, figure
How data was acquired	X-ray photoelectron spectroscopy (XPS) was performed with a VG ESCALAB 3MK II (Thermo VG Scientific), using non-monochromated Al Kα X-rays (*hν*=1486.6 eV), at an instrument resolution of 0.85 eV and a perpendicular take-off angle. The analysis chamber pressure was <10^−9^ Torr. Following Shirley background removal, the component peaks were separated by the VG Avantage software.
Data format	Analyzed, etc.
Experimental factors	The energy was calibrated by setting the C1s C–C peaks of all but the negative SPIONs to 285 eV; the energy of the negative SPIONs was calibrated by setting the more prominent C–Si peak to 284.5 eV. FWHM values were those previously established in our laboratory.
Experimental features	Drops were deposited onto highly oriented pyrolytic graphite (HOPG) and permitted to dry
Data source location	École Polytechnique, Montréal, QC, Canada.
Data accessibility	Data are available with this article

## **Value of the data**

• Demonstration that the symmetric peak analysis of XPS data can characterize the surface chemistry of SPIONs, and their modifications.
• Demonstration of batch-to-batch variations in SPION surface chemistry.
• Demonstration that the water dialysis of SPIONs causes changes in SPION surface chemistry.

## 1 Data

SPIONs, treated with both aminosilane and carboxylic acid silane, were dialyzed to remove contaminants. This apparently mild process was found to modify the SPION surface chemistry. See Table 1 on "A comparative physicochemical, morphological and magnetic study of silane-functionalized superparamagnetic iron oxide nanoparticles prepared by alkaline coprecipitation" [2].

## 2 Experimental design, materials and methods

**Fig. 1:**
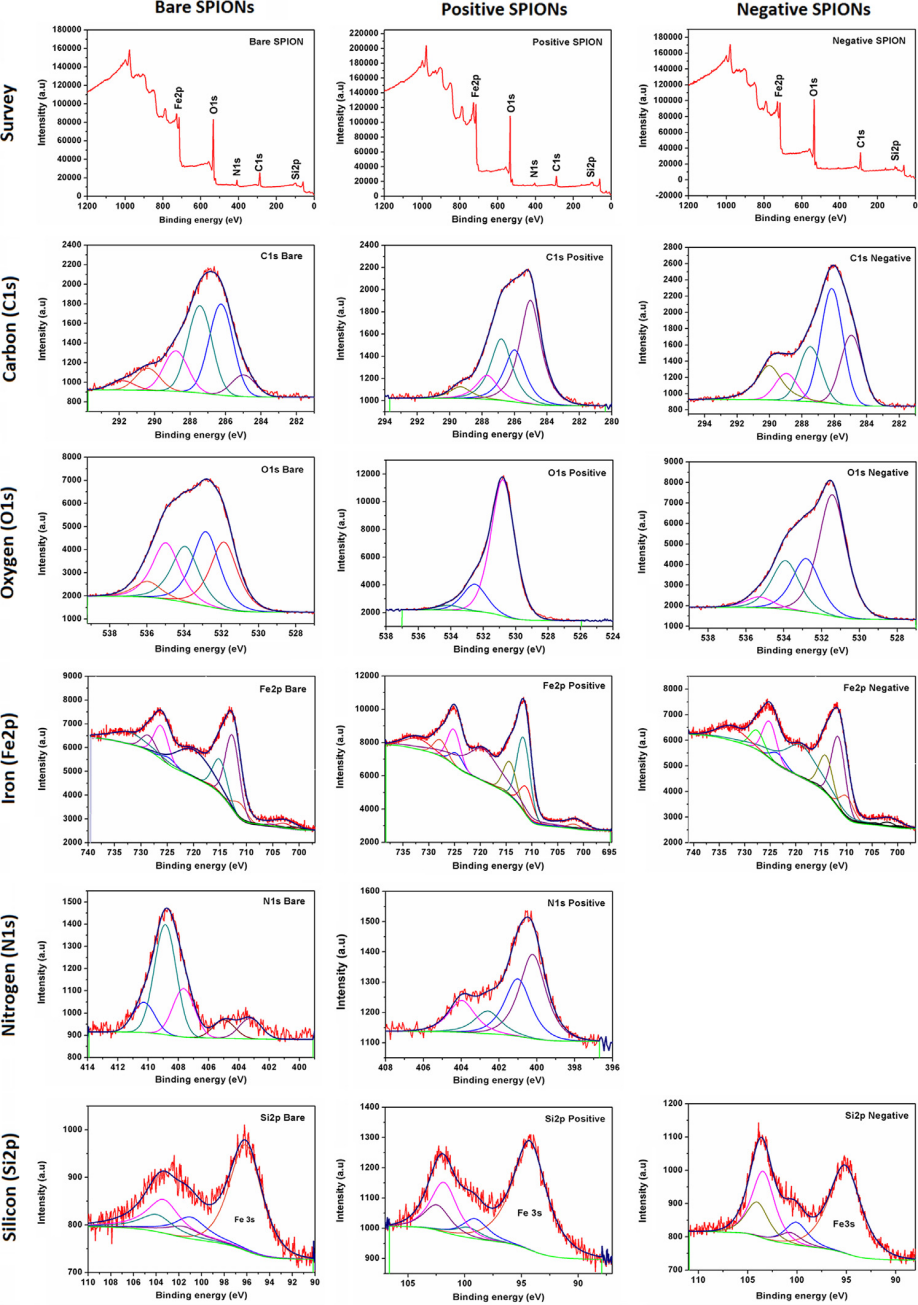
High resolution XPS spectra of positive, negative and bare SPIONs, second batch, before dialysis.

**Fig. 2:**
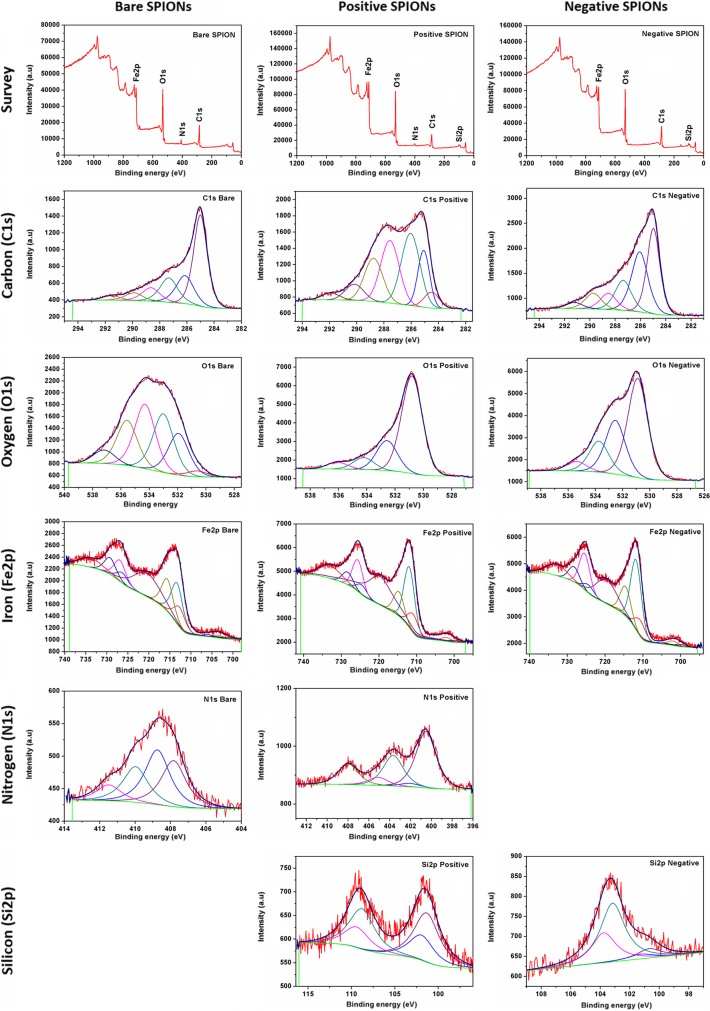
High resolution XPS spectra of positive, negative and bare SPIONs, second batch, after dialysis.

Using a membrane with a 14 kD cutoff, the three SPIONs were each dialyzed for three days, with deionized water being changed several times a day. The peak comparisons of the XPS spectra, before (Fig. 1) and after (Fig. 2) dialysis, are found in Table 1 on "A comparative physicochemical, morphological and magnetic study of silane-functionalized superparamagnetic iron oxide nanoparticles prepared by alkaline coprecipitation" [2], which demonstrates the continued presence of impurities, despite the efforts made to clean the apparatus used, as well as the occurrence of unexpected reactions. These results indicate other sources of batch-to-batch inconsistencies in the manufacture of SPIONs, as we recently noted [2], [3]. Such inconsistencies become important because they determine whether, and to what extent, the surface can be functionalized for use in the human body. The surprising new peaks that appear on dialysis suggest that even this process may provoke some reactions (recall that Fe_3_O_4_ SPIONs are catalysts [4]).

In summary, we have used XPS to characterize the surface chemistry of SPIONs destined for use as prodrugs. The unexpected appearance and disappearance of component peaks demonstrates the apparently unavoidable batch-to-batch differences found on the nanoscale, as well as the usefulness of the XPS technique in determining them. This information is needed even before hemo- and cytotoxicological testing occurs, and demonstrates the serious challenges facing manufacturers of prodrugs.

## Figures and Tables

**Table 1:** Summary of XPS spectral deconvolutions of SPIONs, second batch, before and after dialysis.

**SECOND BATCH – peak position**						
**Suggested attribution**	**Bare SPIONs (eV)**		**Positive SPIONs (eV)**		**Negative SPIONs (eV)**	
**BEFORE DIALYSIS**	**AFTER DIALYSIS**	**BEFORE DIALYSIS**	**AFTER DIALYSIS**	**BEFORE DIALYSIS**	**AFTER DIALYSIS**
C–Si				284.5		
C–C	285.0	285.0	285.0	285.0	285.0	285.0
C–N			286.0			
C–O	286.3	286.1	286.8	286.1	286.2	286.1
C=O	287.5	287.3	287.7	287.6	287.5	287.4
COOH	288.8	288.6 (?)	289.4	288.8	288.9	288.6 (?)
COO-	290.4	289.9		290.1	290.0	289.7
***	291.8	291.5		291.9		291.2
Fe–O		530.6	530.8+Fe–OH	530.8+Fe–OH		530.9+Fe–OH
C=O	531.8+Fe–OH	531.9+Fe–OH	532.5	532.6+O–Si	531.4+Fe–OH	532.5+O–Si
C–O	532.8	533.0			532.8	
C–OH/ O–N		534.3	534.1	534.2	533.9	533.7
***	535.0	535.6		536.1	535.3	535.4
***	536.0	537.2				
						
NH_2_			400.2	400.5		
NH_3_^+^			401.0			
NO			402.6	402.1		
NO_2_	403.3			403.7		
***	405.0		404.0	405.1		
NO_3_ organic	407.7	407.8				
NO_3_ inorganic	408.8	408.7		408.0		
***	410.3	410.0				
***		411.5				
Fe II octa	711.1	711.7	711.0	710.9	710.0	710.7
Fe III octa	712.6	713.6	711.8	711.9	711.6	711.8
Fe III tetra	715.0	716.4	714.2	714.7	714.1	714.6
Si–C			99.2		100.1	
Si–O	101.0			101.3		100.7
Si–O_2_			101.9			
Si–O_3_	103.4				103.4	103.2
***				108.8		

## Appendix A Supplementary material

Supplementary material

The ARC (research contract AUWB-2010-10/15-UMONS-5), the FNRS, the Walloon Region, the COST TD1004 and TD1402, the UIAP VII program and the Center for Microscopy and Molecular Imaging (CMMI, supported by the European Regional Development Fund and the Walloon Region), are thanked for their support.
