# Supplementary material for: Washing effect on superparamagnetic iron oxide nanoparticles
Source: Data Brief. 2016 Apr 7;7:1296–301. doi: 10.1016/j.dib.2016.03.104 (PMC4838932; doi:10.1016/j.dib.2016.03.104)
Supplement: Supplementary file 1 — Supplementary material [file mmc1.zip › conflicts of interest (Signatures) (1).pdf]

This statement is signed by all the authors to indicate agreement that the above information is true and correct (*a photocopy of this form may be used if there are more than 10 authors*):

Author's name (typed)	Author's signature	Date
Laura Karina Mireles Nunez	[illegible]	March 23th 2016
L'Hocine Yahia	[illegible]	March 23th 2016
EDWARD SACHER	Edward Sacher	March 23, 2016
Sophie Laurent	S. Laurent	March 24, 2016
DIMITRI STANICKI	[illegible]	March 24th, 2016
